# Balancing the Double-Edged Implications of AI in Psychiatric Digital Phenotyping

**DOI:** 10.1080/15265161.2023.2296437

**Published:** 2024-01-31

**Authors:** Katherine Bassil

**Affiliations:** a University Medical Center Utrecht; b Maastricht University

Shen et al. (2024) present a new and updated framework on the ethical, social, and legal implications of returning individual research results (IRRs) in digital phenotyping psychiatric research. They highlight a few considerations that have consequences for the returning of IRRs including the ethical, legal, and social sensitivity of the data being collected, uncertainties about the clinical validity and personal utility of the data, and the potential risks to third parties. Additionally, they acknowledge the need to address potential risks in artificial intelligence (AI) and machine learning (ML) systems that are employed to facilitate data collection and interpretation. In this Open Peer Commentary, I will elaborate on the ethical implications arising from integrating AI/ML systems into digital phenotyping, revealing the spectrum of opportunities and risks, particularly in psychiatric research.

Increasingly, AI techniques are being implemented to personal sensor big data with the aim of detecting mood, behavior, and availability of mental disorders (Insel 2017). Shen et al. (2024) caution returning IRRs to participants that were analyzed and interpreted solemnly by ML tools, in the absence of humans in the loop, given increased skepticism surrounding the methods being used and the associated risks of these systems. One of the concerns of AI-based systems relate to bias and fairness within models of behavioral interpretation and prediction (Shen et al. 2024). Non-representative datasets are common and may lead to tools that are less accurate and reliable but could even pose harmful risk to underrepresented groups. Another issue relates to the transparency of the AI systems and their level of explainability to researchers, healthcare providers, research participants and the like. In their article, they argue that despite these risks, their proposed framework does not presume that processed data and interpreted IRRs are always better when conducted by humans versus AI/ML models, with some instances showing the opposite to be preferable (Shen et al. 2024).

Returning IRRs in psychiatric digital phenotyping raises unique ethical considerations, particularly in relation to AI-based analysis of this highly sensitive data. In this commentary, I highlight the double-edged implications of AI-based systems in psychiatric digital phenotyping. One the one hand I explore some opportunities in implementing AI systems, namely AI chatbots, showcasing their capacity to address certain challenges. Conversely, I examine the inherent risks linked to implementing AI systems, potentially amplifying existing ethical dilemmas in this domain.

## EXPLORING AI OPPORTUNITIES

Interpreting digital phenotyping results is associated with several challenges due to the sensitivity, complexity, and volume of data generated. AI and ML can serve as tools to further increase the potential of digital phenotyping and in turn ameliorate the quality of mental health services and support (Li et al. 2023). For example, trained AI algorithms could identify behavioral changes and patterns linked to symptoms of mental illness early on and continuously (Oudin et al. 2023). The application of AI extends beyond the analysis and interpretation of large digital phenotyping datasets for returning IRRs. Several other opportunities exist to introduce AI and ML at different stages of the digital phenotyping process.

For instance, the shortage of digital phenotyping counselors—who play an important role in assessing digital phenotyping results, could be addressed by the implementation of AI chatbots. AI chatbots allow accessible support for research participants by providing immediate guidance, insights, and feedback based on the analyzed data (Khawaja and Bélisle-Pipon 2023). Their continuous availability offers a scalable solution to bridge the gap between the demand for counseling and the limited availability of human counselors, ensuring timely and consistent assistance for research participants undergoing digital phenotyping assessments. AI chatbots could provide assessment support based on the data, immediate guidance in the form of alerts, personalized feedback and support, and educational resources to improve the participant’s understanding of their data patterns and information for mental health support.

Additionally, as Shen et al. (2024) argue, the language employed to return IRRs to participants could highly influence the ability to comprehend or misunderstand the information, and as such potentially increase the associated risks. The implementation of AI creates the opportunity to overcome this challenge by providing individuals with AI-generated responses, for instance through personalized AI chatbots, that are trained to reflect the participant’s own native language and communication preferences. For example, when the response language of chatbots was manipulated to match users’ dominant personality type, a positive improvement in user engagement and interaction was observed (Shumanov and Johnson 2021).

## NAVIGATING AI RISKS

The use of AI in digital phenotyping is associated with a number of ethical risks. Shen et al. (2024) highlight the issue of bias and fairness in the implementation of AI when returning IRRs. However other concerns including privacy and data protection, consent, and accountability, have been previously discussed (Martinez-Martin, Greely, and Cho 2021). The implementation of AI chatbots in digital phenotyping also comes with ethical implications that require scrutiny, particularly in the context of psychiatry and mental health. One of the biggest concerns for participants engaging with mental health applications through digital phenotyping is attributed to the disclosure of personal and sensitive information (Martinez-Martin, Greely, and Cho 2021). That is why, addressing these ethical issues is paramount given this growing concern among participants and users.

The deployment of AI chatbots as digital phenotyping counselors for instance, could raise questions regarding the validity and safety of these tools. Particularly in the case of unsupervised chatbots, where the accuracy of responses and their adherence to ethical guidelines might come under scrutiny (Coghlan et al. 2023). The absence of human oversight (in the form of experts) in unsupervised chatbots could potentially lead to unpredictable interactions and the potential sharing of misinformation or inadequate support, accentuating concerns about the reliability and ethical standards of these AI-driven counseling tools. This is particularly concerning, given that chatbots can be designed (e.g., personalized response language) in ways that could influence user behavior, with potential room for manipulation and misuse (Shumanov and Johnson 2021).

Moreover, one of the ethical concerns lies in the potential of AI utilized in analyzing and interpreting digital phenotyping results to prioritize clinical utility over personal utility. This bias could result in the user being unable to access or act upon insights crucial to their personal meaning and personal decision-making, and as such sidelining information they consider pivotal for their well-being or personal preferences. Personal meaning has been repeatedly shown to be an important factor that needs to be taken into consideration when it concerns navigating mental health problems (Arslan, Yıldırım, and Leung 2021; Ravitsky and Wilfond 2006). There remain questions as to how the AI algorithms will be designed and trained in a way to balance between clinical and personal utility in ways that reflect respect for autonomy, maximize the benefits and minimize potential harms and risks for users.

## CONCLUSION

Shen et al. (2024) present a framework focused on the ethical, legal, and societal aspects of returning IRRs in digital phenotyping research. They mention some of the ethical implications that are associated with integrating AI and ML systems for analyzing and interpreting these results, mainly issues related to bias, representation, and fairness. However, there exists several other opportunities and risks that accompany AI-generated responses in the form of analyzed results, alerts, or conversations in psychiatric digital phenotyping. One of these opportunities involve the introduction of AI chatbots as digital phenotyping counselors, and AI-based personalized response languages to provide improved engagement and experience with the IRRs. Of the associated risks, the lack of predictability and implications of (unsupervised) AI chatbots, in addition to the possibility of undermining users’ personal utility could negatively impact the reliability and ethical standards of AI-based digital phenotyping systems.

Finally, the opportunities and risks outlined in this article align well with Shen et al.’s (2024) proposed framework for returning IRRs, highlighting its adaptability in accommodating the ongoing emergence of risks associated with integrating disruptive technologies like AI into digital phenotyping and similar applications. This in turn, showcases the framework’s flexibility in addressing the dynamic landscape of challenges within this evolving field. Future research aimed at thoroughly probing the ethical implications associated with implementing AI within psychiatry and mental health contexts is needed. Understanding and addressing these implications are imperative to ensure responsible and ethical deployment of AI technologies, safeguarding individual’s well-being and preserving trust in these evolving applications.
